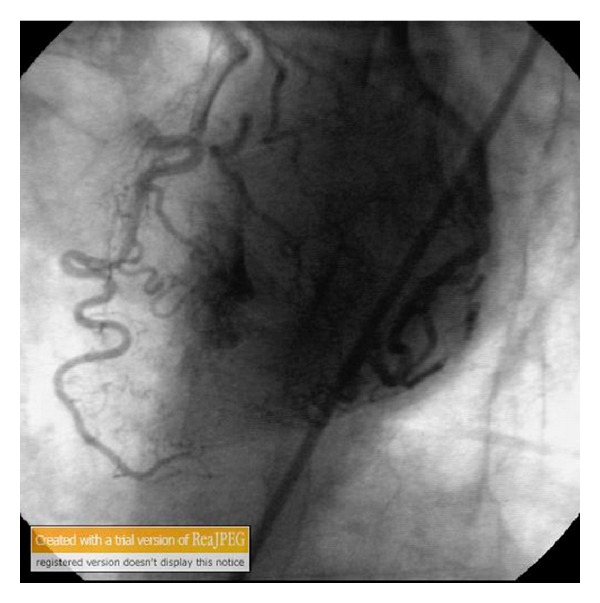# First Diagonal Coronary Artery: Left Ventricular Fistula Presenting as Unstable Angina

**DOI:** 10.1155/2013/908162

**Published:** 2013-08-12

**Authors:** Murat Sener, Mehmet Akkaya, Muammer Bilici

**Affiliations:** ^1^Department of Cardiology, Siirt Devlet Hastanesi, 56000 Siirt, Turkey; ^2^Department of Internal Medicine, Siirt Devlet Hastanesi, 56000 Siirt, Turkey

## Abstract

Coronary artery fistulae are characterized by communications between a coronary artery and a cardiac chamber or another vascular structure. They are usually congenital, but acquired forms may occur. Most patients are usually asymptomatic. However, some studies have emphasized that the incidence of symptoms and complications increases with age, particularly after the age of 20 (Liberthson et al. 1979, Hong et al. 2004). We aimed to present a very rare form of fistula originating from the first diagonal artery and connecting into the left ventricle.

## 1. Case Report

### 1.1. History

A 76-year-old woman with a history of hypertension and hyperlipidemia was admitted to our emergency department with typical angina pectoris ongoing for 30 minutes. On admission, her arterial blood pressure was 160/95 mmHg; pulse rate was 72 bpm. Her physical examination was normal. The 12-lead electrocardiogram revealed normal sinus rhythm, left ventricular hypertrophy (27 mm R wave V6), and T wave inversion in leads II, III, aVF, I, aVL, and V3–V6. The chest X-ray showed no cardiomegaly or pulmonary congestion. Cardiac enzymes were not raised throughout the admission. Echocardiography revealed normal ventricular function without wall motion abnormality and left ventricular hypertrophy.

### 1.2. Angiography

**Figure 1:**
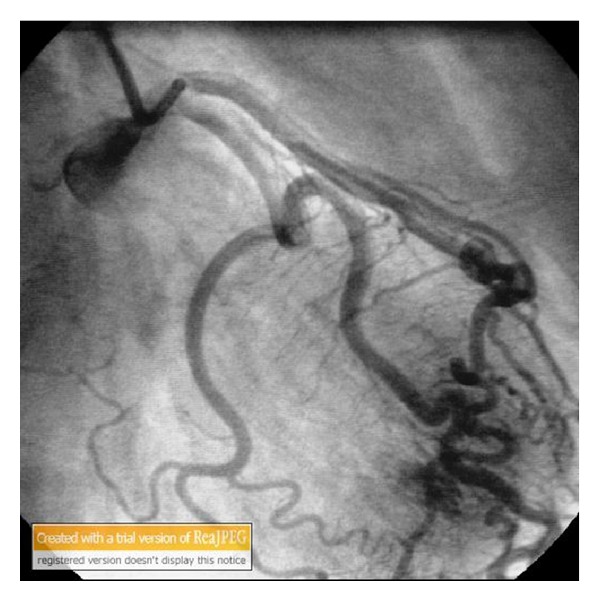


**Figure 2:**
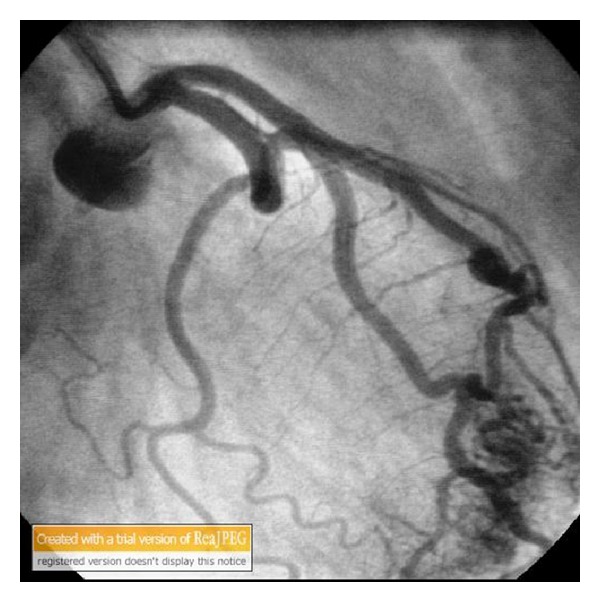


1. Figures 1, 2, and 3 show the fistula from the first diagonal artery to the ventricle. There was no stenosis in the coronary arteries (Figures 1, 2, and 3).
2. Left ventriculography demonstrated good systolic function (EF = 60%) without wall motion abnormality.

## 2. Discussion

This infrequent abnormality is an incidental finding in 0.3–0.8% of adult population referred to for coronary angiography [1–4]. Coronary artery fistulas can occur from any of the three major coronary arteries as well as the left main trunk [5]. The right coronary artery or its branches are the site of the fistula in about 55% of cases, the left coronary artery in about 35%, and both coronary arteries in 5% [6]. Circumflex coronary artery involvement is uncommon. Fistulous drainage occurs into the right ventricle in 41%, right atrium in 26%, pulmonary artery in 17%, left ventricle in 3%, and superior vena cava in 1% [7]. Iatrogenic causes should be considered including surgery and percutaneous intervention. We presented this case because a fistula from the diagonal artery to the left ventricle is very rare, particularly in patients with advanced age. In our patient, because of her advanced age, we recommended drug treatment after which she was asymptomatic.

ECG abnormalities are usually due to left atrial enlargement, left ventricular hypertrophyenlargement, or myocardial ischemia. In this case ECG features were attributed to left ventricular hypertrophy.

The optimal therapeutic strategy for coronary artery fistulas is not clear. The best management strategy for asymptomatic patients is observation due to that the incidence of late complications is very low. However, some authors advocate fistula closure even in asymptomatic patients for prevention of complications because of the high success rate and low risk of complications [2, 8]. The main indications for closure are (1) clinical symptoms, especially of heart failure and myocardial ischemia, and (2) in asymptomatic patients with high-flow shunting, to prevent the occurrence of symptoms or complications, especially in the pediatric population. Surgical correction is safe and effective with good results [9, 10].

## Figures and Tables

**Figure 3:**